# On-Demand Charging Management Model and Its Optimization for Wireless Renewable Sensor Networks

**DOI:** 10.3390/s22010384

**Published:** 2022-01-05

**Authors:** Sandrine Mukase, Kewen Xia, Abubakar Umar, Eunice Oluwabunmi Owoola

**Affiliations:** 1 School of Electronics and Information Engineering, Hebei University of Technology, Tianjin 300401, China; ssandrinem@gmail.com (S.M.); 201940000018@stu.hebut.edu.cn (E.O.O.); 2 School of Mechanical Engineering, Hebei University of Technology, Tianjin 300401, China; 201541201002@stu.hebut.edu.cn; 3 Research and Product Development Department, Nigerian Institute for Oil-Palm Research, Benin City 300001, Nigeria

**Keywords:** energy consumption, particle swarm optimization, wireless energy transfer, energy renewable management system, wireless renewable sensor networks

## Abstract

Nowadays, wireless energy transfer (WET) is a new strategy that has the potential to essentially resolve energy and lifespan issues in a wireless sensor network (WSN). We investigate the process of a wireless energy transfer-based wireless sensor network via a wireless mobile charging device (WMCD) and develop a periodic charging scheme to keep the network operative. This paper aims to reduce the overall system energy consumption and total distance traveled, and increase the ratio of charging device vacation time. We propose an energy renewable management system based on particle swarm optimization (ERMS-PSO) to achieve energy savings based on an investigation of the total energy consumption. In this new strategy, we introduce two sets of energies called emin (minimum energy level) and ethresh (threshold energy level). When the first node reaches the emin, it will inform the base station, which will calculate all nodes that fall under ethresh and send a WMCD to charge them in one cycle. These settled energy levels help to manage when a sensor node needs to be charged before reaching the general minimum energy in the node and will help the network to operate for a long time without failing. In contrast to previous schemes in which the wireless mobile charging device visited and charged all nodes for each cycle, in our strategy, the charging device should visit only a few nodes that use more energy than others. Mathematical outcomes demonstrate that our proposed strategy can considerably reduce the total energy consumption and distance traveled by the charging device and increase its vacation time ratio while retaining performance, and ERMS-PSO is more practical for real-world networks because it can keep the network operational with less complexity than other schemes.

## 1. Introduction

Embedded electronics and wireless devices are rapidly developing, and wireless sensor networks (WSNs) have attracted significant attention among the research community. Wireless sensor networks are growing rapidly in industry, where intelligent gadgets are used to monitor a system’s intelligence, and in academic research, as a latent research domain. Wireless sensor networks are typically made up of tiny devices known as sensor nodes (SN), which have limited computational capability due to their limited battery capacity. Given the limited battery capacity of nodes, lifetime and energy consumption are critical hindrances that prevent their proper deployment. To improve energy routing protocols, different previous studies have often reduced the energy consumption of sensor nodes [1]; some have employed media access control (MAC) protocols [2] or topology control protocols [3] to minimize energy usage by elongating the network lifespan. However, they cannot completely solve the energy bottleneck for the deployment of the wireless sensor network. Therefore, it is essential to restore the energy of sensor nodes to equilibrate and extend the network lifetime [4]. At present, there are three categories of solutions existing for the energy replenishment of the sensor nodes, namely energy harvesting [5], wireless energy transfer (WET) [6] and sensor node replacement [7]. Several researchers have used energy harvesting techniques to empower the energy supply towards the sensor nodes, such as solar energy [8] and wind energy [9]. However, due to the variations and changes in the natural environment, the collection rate of energy harvesting techniques is difficult to predict, and its time-varying nature makes it unreliable. Moreover, the replacement of the sensor node is either expensive or it is impracticable due to the dangerous environment. The wireless energy transfer technology has made a breakthrough, by resolving the energy bottleneck of wireless sensor networks and extending their lifetime [10]. The combination of wireless energy transfer and a wireless sensor network is called a wireless renewable sensor network (WRSN). A wireless rechargeable sensor network concerns a WSN that is replenished by wireless energy for sensor nodes using wireless energy transfer techniques.

In Ref. [11], the most recent development in WET technology provides valuable knowledge for powering sensor nodes. In contrast to other energy harvesting strategies, WET has several advantages, including immunity from a strict source and receiver management, highly efficient energy transfer, resistance to environmental circumstances (dirt, chemicals and air) and small device size [12]. One of the classic solutions to the mobile charging problems is the traveling salesman problem (TSP) charging protocols [13]. Periodically, the wireless mobile charging device carries out the charging techniques following a pre-optimized tour that helps to charge the sensor nodes in a short period with a small WMCD travel distance. However, when the nodes’ energy consumptions are dissimilar, the TSP-based solution may result in unnecessary visits to nodes with sufficient energy. When mobile charging devices perform sensor node charging tasks, this challenge not only augments the charging device travel distance but also increases the waiting time before low-energy nodes can be charged. Unlike previous schemes [13,14,15], where a wireless mobile charging device (WMCD) charges all sensor nodes in a renewable cycle, in our strategy, a WMCD only visits and charges a small group of SNs in each cycle.

The major goal is thus to reduce node energy consumption to keep all nodes operational, as well as to minimize the WMCD travel distance, which will increase its vacation time. This is referred to as network eternal functioning, and it is one of the key aims in the design of WRSNs. Seeking an ideal method to schedule a fleet of WMCDs for recharge is typically an NP-hard problem, whereas traditional efforts using standard optimization approaches are not cost-effective due to the WMCD’s limited computing resources. Thus, in practice, heuristic algorithms are typically recommended to establish a good compromise between optimality and computing complexity.

Wireless energy transfer is an energy harvesting method to address the network’s lifetime bottleneck. Kurs et al. [11] considered the development of recharging performance in the WSN by successfully illuminating a 60 W bulb over a distance of two meters using magnetic resonance. Several models for wireless energy transfer have been proposed, each with a different specific goal. Dai et al. [16] suggested a near-optimal solution to the problem of safe charging in a sensor network deployment with a static base station. The suggested scheme reduces the unexpected emission of electromagnetic radiated waves from several sinks to a predefined level. One of the challenges that must be addressed is the selection of an ideal point for electromagnetically radiated signals in a plane. They demonstrated that rechargeable sensors can harvest energy from other sources and maximize the charging efficiency.

Zhang et al. [17] proposed a scheme called ERSVC, and, in their strategy, they prove that the traveling distance plays a large role in energy consumption. To save energy in ERSVC, they created a detailed plan for data flow, charging duration and visiting sets. Furthermore, they showed that ERSVC can theoretically ensure that the network operates forever. However, their method of charging nodes in each cycle is still a problem because they wait for their sensor nodes to approach the general minimum energy level, and this can cause the WMCD to consume more time and energy, reduce its vacation time and cause nodes to start dying in early rounds. A dynamic optimal scheme was proposed in [18] that aimed to increase the vacation time ratio of a wireless mobile charger. In their work, they offered a novel method for determining the best placement of the service station. To ensure enough coverage, not all sensor nodes in each subnetwork are chosen as active nodes. When the sensor node’s remaining energy for active nodes falls below a certain threshold, the sensor node stops working and waits for charging.

Zhao et al. [19] showed how to schedule charging and allocate charging time simultaneously by prolonging the network lifetime and improving the charging efficiency using a proposed mixed-integer optimization algorithm. According to the simulation results, the suggested algorithm may achieve a charging success rate of 100 percent in periodic and hybrid services, which is much higher than the comparison methods. However, due to the enormous number of request nodes in large-scale networks, it is difficult for a single vehicle to meet all charging requests. Zhong et al. [20], in the context of incomplete mobile charger device capability, presented a real-time, on-demand charging scheduling scheme (RCSS) that examined the dynamic energy consumption of different nodes when creating the charging path and proposed the next node selection method. Simultaneously, a method for determining the feasibility of charging circuits was suggested to ensure charging efficiency and was based on an adaptive charging threshold through the charging procedure to decrease node death

In Ref. [21], the authors started with a Linear Programming (LP) approach to solve the problem of scheduling a mobile charger, and they then moved on to an efficient scheme based on the gravitational search algorithm (GSA). Their solution consists of a novel agent perception as well as an effective fitness function. They ran extensive simulations on the proposed system to demonstrate how it outperforms two cutting-edge algorithms: first come, first served (FCFS), and nearest job next with preemption (NJNP). To extend the lifetime of sensors, researchers have used mobile chargers and external energy sources [22]. In their research, they looked at periodic charging time scheduling and charging path planning with multiple chargers. They described a slot-based periodic recharge time scheduling algorithm that includes a fine-grained node classification strategy to avoid wasted trips to energy-sufficient nodes and a balanced charging task assignment scheme to avoid charging famine. They also developed a charging path planning algorithm that allows for parallel energy replenishment using multiple chargers, which resulted in higher charging efficiency.

The energy capacity issue can be solved using a wireless energy transfer (WET) technique (WRSNs). Dong et al. [23] proposed a demand-based charging mechanism (DBCS) for wireless renewable sensor networks by improving charging programming in different ways: clustering approach, charging node selection, charging trail and charging timetable. Firstly, they presented a multipoint improved Kmeans (MIKmeans) clustering system that can group nodes based on their location, remaining energy and past contribution to balance energy usage. Secondly, the dynamic selection algorithm for charging nodes (DSACN) was developed to select nodes that need to be charged. Finally, they presented the DBCS to improve the efficiency of the mobile charging vehicle and developed simulated annealing based on performance and efficiency (SABPE) to minimize the charging time and path distance of the charging vehicle.

Kennedy and Eberhart introduced Particle Swarm Optimization (PSO) in (1995), which is a randomly global swarm-based intelligence algorithm [24]. PSO is determined by the common behavior of animals such as a group of birds, a school of fish that protects against predators or a swarm of bees looking for food sources. Particle swarm optimization also has been used for scheduling charging models in wireless rechargeable sensor networks. To improve the wireless charging vehicle’s journey time, the cost of the charger’s travel path between nodes must be considered; Dhurgadevi et al. [22] presented a PSO-based heuristic for scheduling the journey path of a wireless charging vehicle that considers both travel cost and travel time. The Particle Swarm Charger Deployment (PSCD) and Improved PSCD (IPSCD) algorithms are introduced in [24] to almost optimize WRSN charger deployment by utilizing the particle swarm optimization (PSO) concept. Distances between chargers and nodes that were the same as the angles between them were used to calculate the charging efficiency in PSCD and IPSCD. To acquire statistics on charging efficiency, the authors conducted experiments using real-world wireless chargers.

PSO has a fast convergence speed, which is best for wireless sensor network applications. However, this fast convergence speed can result in several drawbacks, including poor global search ability, low accuracy, fast convergence and stagnation. Reference [25] observed that PSO is quick at locating a promising region, but cannot refine the solution. Exploration refers to the characteristics of particle swarm optimization that allow it to describe favorable regions in the search space, whereas exploitation refers to the ability of the PSO algorithm to enhance solutions within these favorable regions. Extensive studies show that PSO’s ability to find a global optimum mostly depends on exploration and exploitation tendencies [26,27,28]. Exploration is beneficial for a multimodal search but compromises convergence speed in the multimodal search, while exploitation is more beneficial for a unimodal search. The contributions of [26,27,28,29] demonstrated that these properties could be adjusted by carefully choosing the value of inertia weight (ω) in the particle swarm optimization algorithm. The standard PSO is the version of the PSO that is used today. Techniques that have been successfully implemented for modifying the algorithm include enhanced strategies [30], hybrid strategies [31,32,33] dynamic neighborhood topology [34,35,36] and time-varying parameters [37,38,39,40], etc.

Our paper is the first that aims to solve this problem by investigating the mobile charging request strategy called the energy renewable management system, based on particle swarm optimization (ERMS-PSO), and we examine four metrics together in parallel, which are vacation time (of WMCD) efficiency, the total distance traveled by the WMCD, the total energy consumed by the WMCD by traveling through the target nodes and the total number of cycles.

Unlike [17] during each cycle, where they considered the maximum energy (Emax) and minimum energy (Emin) in the nodes and optimized the node’s energy consumption and vacation time ratio, for our strategy, we took into account the total power consumption, total distance traveled and wireless mobile charging device’s vacation time ratio as performance metrics and aimed to investigate the mobile charging request strategy called the energy renewable management system, which is based on particle swarm optimization (ERMS-PSO). In this new strategy, we introduced two sets of energies, called emin (our minimum energy) and ethresh (our threshold energy), where, when the first node reaches our emin, it will inform the base station (BS), also known as the sink node, which is responsible for collecting all information from sensor nodes and sending it to the end-user. The base station will calculate all nodes’ energy that fall under ethresh and send a WMCD to change them in one cycle. Our emin must be greater than the Emin (general minimum energy in the node) and our ethresh must be greater than emin but less than the general Emax.

The following are the main contributions and innovations of this paper:

1. First, we investigate the operation of a sensor network and propose an on-demand energy-saving strategy called the energy renewable management system (ERMS) for keeping the network operational for a long time by examining the wireless mobile charging device vacation time efficiency, the total distance traveled by the WMCD, the total energy consumed and the total number of cycles.
2. Secondly, based on the presented strategy, a heuristic algorithm called particle swarm optimization (PSO) is developed and successfully implemented for solving the energy replenishing problem of the wireless sensor network and developing a suitable fitness function for achieving the said objectives.
3. This work aims to solve the problem of wireless energy transfer by investigating the mobile charging request strategy, where two sets of variables are introduced—emin, ethresh—to help manage the energy in the node and levels of charging of WMCD. We finally compare the results from the proposed algorithm with other notable algorithms.

We propose a charging scheme that can improve the current designs while decreasing the effect of their limits.

The remaining part of this work is arranged as follows. We describe the problem statement in Section 2 and we analyze the total energy consumption in Section 3. Section 4 explains in detail the proposed strategy, ERMS-PSO. Results are delivered in Section 5, with comparison obtained through both experiments and simulations, and we conclude and give suggestions for future work in Section 6.

## 2. Problem Description

In this section, we describe the model of WMCD behavior as well as the WSN control approach. Abbreviations contain a list of the main acronyms and notations used in this paper.

The energy harvesting method not only increases the lifespan of individual sensor nodes but also the overall lifespan of the WSN. We take into account a two-dimensional area with N randomly distributed sensor nodes, which is similar to the scenario used in [13,14,15]. Each node has an energy receiving device that allows it to recharge its batteries remotely. A wireless mobile charging device (WMCD) has a static base station (BS) and a rest station (RS), also known as a service station, which is a place that the mobile charger uses to rest and recharge its battery for the next tour.

**Figure 1:**
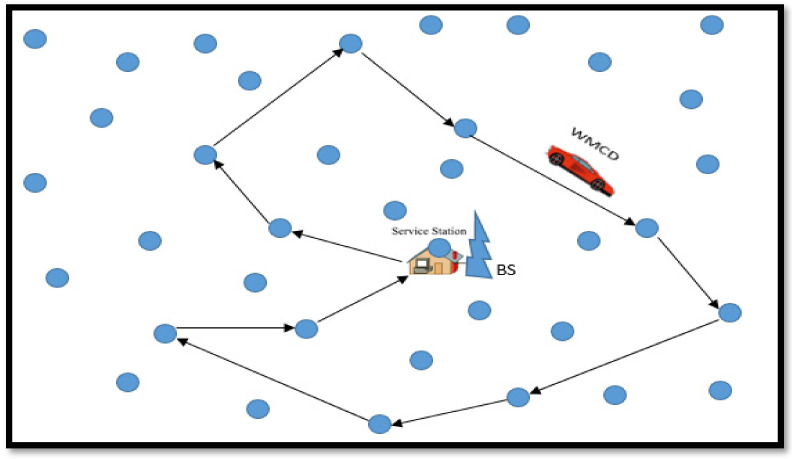
A WSN with a wireless mobile charging device.

The WMCD renewable energy cycle is known as the Hamiltonian cycle, and it begins and finishes at RS, as seen in Figure 1. The WMCD battery, on the other hand, is recharged on the RS via immobile energy storage and takes the lead from the RS, proceeding to the nearest node. The WMCD process continues as all deployed nodes use WET to wirelessly recharge their batteries. It returns to its rest station after recharging the node batteries to replenish its battery for the next charge cycle. A wireless renewable sensor network (WRSN) is defined as a WSN built with a WMCD. In real world scenario, The WMCD could be a vehicle following a set path.

The WMCD must visit and charge some sensor nodes during each cycle (C). *H_n_* denotes the group of nodes that must be visited in the *Cth* cycle. WMCD goes through the smallest Hamiltonian cycle that joins all nodes in *H_n_* and the RS in the *Cth* cycle. *P_n_* represents the shortest Hamiltonian cycle’s traveling path. *D_n_* denotes the length of path *P_n_*, and *t_n_* denotes the time spent traveling across distance *D_n_*.

*T* is denoted as the time required for the WMCD’s tour cycle and *t_vac_* is the vacation time of the WMCD in the *Cth* cycle. During the *Cth* cycle, the WMCD travels from RS to *H_n_*, visiting and charging nodes before returning to RS for *t_vac_*. The cycle time *T* is calculated as follows:

(1) $$T=t_{n}+t_{vac}+\underset{j\in h_{n}}{\sum }t_{j}$$

(2) $$t_{n}=\frac{D_{n}}{V}$$

where $\sum_{j\in h_{n}}t_{j}$ is the entire length of time that the WMCD spends in charging all nodes in *H_n_*.

We employ a periodic strategy in this paper. As previously stated, a WMCD is used to charge the sensor nodes periodically on a *T*-cycle basis. Each node, on the other hand, should be charged on a regular basis to complement its energy use. Previous studies [13,14,15] found that the WMCD goes into the network and charges all nodes in every cycle. In real-world networks, however, sensors’ energy consumption rates may vary. Some nodes near the base station may spend several times less energy than faraway nodes; therefore, it is not necessary to charge every node in the cycle.

This observation introduced a new research direction in wireless energy transfer research. The questions arising include: Do all nodes need to be charged at every charging cycle? If not, then how do we select the nodes to be charged at any given time? Must the energy in the node approach Emin before charging can be initiated? If not, then what energy level should be initiated?

**Figure 2:**
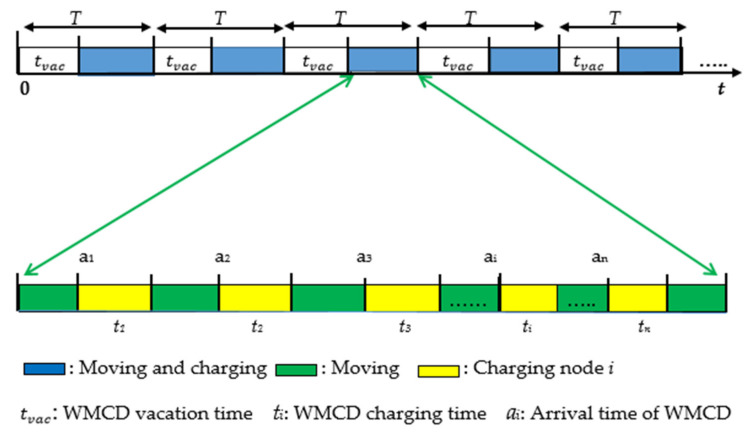
Periodic charging diagram.

As a result, we can use a variety of strategies that consider each node’s energy consumption rate. For example, in each cycle, the nodes with the highest energy consumption will be visited, and those with the lowest amount of energy consumption can be visited every two cycles or more. As shown in Figure 2, the charging procedure for node *i* (*i* ∈ N) should be replenished regularly to supplement its energy usage over time.

The energy conservation principle states that node *i*’s energy consumption should be equal to the WMCD’s energy supply [12].

(3) $$T_{i}*P_{i}=t_{i}*U\;\left(i\in N\right)$$

where *T_i_* represents the visiting and charging interval of *i* and *p_i_* represents the energy consumption rate of node *i*.

## 3. Energy Consumption Analysis

In this section, we use the WMCD to calculate the system’s total energy consumption. To compare, a hybrid vehicle (PHEV) plug-in is used to transport the wireless mobile charging device battery. According to Pacific Northwest National Laboratory research data [41], a mid-size PHEV’s energy consumption per mile (ECPM) is 675 J/m. If the WMCD travels at *V* = 5 m/s, the WMCD’s energy consumption while traveling is approximately 3 kW. The sensor node’s energy consumption, on the other hand, ranges from a few milliwatts to hundreds of milliwatts [42]. The energy required by the WMCD for traveling appears to encompass the majority of the system’s total energy consumption. We introduce a particle swarm optimization-based energy renewable management system (ERMS-PSO). In ERMS-PSO, we first set *T* accurately, and we then calculate the charging period *T_i_* for sensor node *i* based on its energy consumption rate *pi*. As a result, the WMCD should visit a subset of nodes in each cycle, reducing the WMCD’s journey travel distance. We employ two key steps to reduce the two portions of overall energy use. We wish to reduce the WMCD travel distance as much as possible because it accounts for the majority of the total energy use.

- The total power consumption has two parts; their sum is seen in Equation (4) and can be calculated as follows:
  (4) $$P_{total}=\frac{1}{\lambda }*\sum_{i,i\in N}p_{i}+\frac{D_{total}*ECPM}{T_{total}}$$
  where *λ* is the energy conversion efficiency of non-radiative energy transfer. $T_{total}$ is the total time and $D_{total}$ is the distance traveled by the WMCD throughout all cycles.
- The ratio of vacation time of the WMCD ($\mu_{vac}$), which serves as the optimization goal in [13,14,15]. We describe $\mu_{vac}$ in this study as the mean percentage of time in each cycle that the WMCD spent on vacation, and it can be calculated as follows:
  (5) $$\mu_{vac}=\frac{\sum_{k}t_{vac}}{T_{total}},\;\mu_{vac}\in \left[0,1\right]$$
  where $\sum_{k}t_{vac}$ is the total amount of time that the WMCD spends on vacation across all cycles. Our goal in this research is to reduce the value of $P_{total}$. In Equation (5), we can observe that when $\mu_{vac}$ grows, the WMCD has a longer period to repair or replenish its battery at the RS, indicating greater network performance.

## 4. Implementation of Proposed ERMS-PSO

### 4.1. Energy Renewable Management System (ERMS)

ERMS implementation consists of two primary steps. The initial stage is to optimize the first part of the total energy usage $\sum_{i,i\in N}p_{i}$ Secondly, we create a collaborative design to reduce the WMCD’s trip distance.

The flow rate from node *i* to node *j* is represented by *W_ij_*, the flow rate from node *i* to base station BS is represented by *W_iB_*, and *W_ki_* represents the bit flow rate coming from node *k* to *i* and. The flow balance constraint is then applied to each node *i* (Equation (6)).

(6) $$\sum_{k\in N}^{k\neq i}w_{ki}+R_{i}=\sum_{j\in N}^{j\neq i}w_{ij}+w_{iB}$$

where $\sum_{j\in N}^{j\neq i}w_{ij}+w_{iB}$ represents the data flow rate of energy transmission from node *i* to *j* or the base station, and $\sum_{k\in N}^{k\neq i}w_{ki}$ is the data flow rate of energy reception from node *k* to *i*. To transmit and receive data, each sensor node consumes energy. We use the energy consumption model [16] in this article, which has been widely used in previous studies [12,13,14].

(7) $$p_{i}\left(t\right)=\rho \sum_{k\in N}^{k\neq i}w_{ki}+\sum_{j\in N}^{j\neq i}v_{ij}\cdot w_{ij}+v_{iB}\cdot w_{iB}$$

where *ρ* is the constant coefficient, and *V_ij_* and *V_iB_* are the energy consumption for transmitting a unit of data from node *i* to node j or the base station, respectively, which is expressed as follows:

(8) $$V_{ij}=\beta_{1}+\beta_{2}\cdot d_{ij}^{\alpha }$$

Here, $\beta_{1}$ is a distance-independent constant term and $\beta_{2}$ is a distance-dependent term coefficient, *d_ij_* describes the distance between nodes *i* and *j*, while *α* is the path-loss index and is 2 ≤ *α* ≤ 4. We can develop a non-linear function of the BS position (*X_B_*, *Y_B_*) from Equation (7):

(9) $$V_{iB}=\beta_{1}+\beta_{2}{\left[\sqrt{{\left(X_{B}-X_{i}\right)}^{2}+{\left(Y_{B}-Y_{i}\right)}^{2}}\right]}^{\alpha }$$

The energy consumption rate for reception in this model is $\rho \sum_{k\in N}^{k\neq i}w_{ki}$, while the energy consumption rate for transmission is $\sum_{j\in N}^{j\neq i}v_{ij}\cdot w_{ij}+v_{iB}\cdot w_{iB}$ We assume that the network’s flow rates (*W_ij_* and *W_iB_*) are time-invariant. The optimization goal is to minimize the total energy consumption of nodes (i.e., $\sum_{i,i\in N}p_{i}$), which is the first part of *P_total_* in Equation (4). Each node must meet the basic flow balancing constraint in Equation (6), as well as the energy consumption model in Equation (7). As a result, the optimal problem can be expressed as a linear programming problem as follows:

(10) $$\begin{array}{ll} \min & \sum_{i,i\in N}p_{i}\\ s.t. & \sum_{k\in N}^{k\neq i}W_{ki}+R_{i}=\sum_{j\in N}^{j\neq i}W_{ij}+W_{iB}\;\;\;\;\left(i\in N\right) \end{array}$$

(11) $$p_{i}\left(t\right)=\rho \sum_{k\in N}^{k\neq i}w_{ki}+\sum_{j\in N}^{j\neq i}v_{ij}\cdot w_{ij}+v_{iB}\cdot w_{iB}$$

$$W_{ij}\geq 0$$

The optimization variables in this problem are *W_ij_*, *W_iB_* and *p_i_*, while constants are *R_i_*, $\rho ,\;v_{ij}$, and $v_{iB}$. After solving the optimization problem in Equations (10) and (11), the energy consumption rate *pi* can be determined for each node *i*. In this section, we develop a system that combines the charging period *T_i_*, the visiting set *Cth* in each cycle and the WMCD traveling path.

This work aims to solve the problem of WET by investigating the mobile charging request strategy, where two sets of variables are introduced: *e_min_* and $e_{thresh}$. If the maximum energy in the battery of nodes is *E_max_* (usually 10.8 KJ) and the minimum energy is *E_min_* (usually 0.05 × *E_max_*), then the *e_min_* is a low battery indicator, which must be higher than the *E_min_* value, at which the nodes eventually die. The value of *e_min_* is carefully selected to ensure that the nodes in WSN never approach *E_min_*. The value ethresh is the allowable threshold of energy, which must be higher than the emin but lower than the *E_max_*. *E_max_* is a safe charging value that ensures the continuous lifetime of the network while minimizing the charging time.

By communication, when the first node reaches the low battery energy level $e_{min}$ in Equation (12), it initiates a charging request for the WMCD; then, the base station queries all the nodes and identifies nodes with battery energy less than the allowable energy threshold $e_{thresh}$ (Equation (13)); these nodes are lined up as target nodes. The WMCD is required to visit these target nodes in the particular charging cycle. Reference [13] showed that the TSP charging route improves the energy efficiency of the WMCD, but it is believed that this increases the waiting time of the nodes to be charged because some nearby nodes may possess more battery life while some far-away nodes are at the verge of being extinguished. When the WMCD visits each of the target nodes, it charges the battery in the node to the $E_{max}$. Our two energy variables are calculated as follows:

(12) $$e_{min}=E_{min}+\left(E_{max}-E_{min}\right)*X_{1}$$

(13) $$e_{thresh}=E_{min}+\left(E_{max}-E_{min}\right)*X_{2}$$

$$0\leq X_{1},\;X_{2}\leq 1$$

These particular recharging thresholds $0\leq X_{1},\;X_{2}\leq 1$ are shared by all network nodes. If *P_max_* and *P_min_* are the energy consumed by the node with the highest data rates and the lowest data rates, respectively, then the minimum and maximum charging time in a single operating cycle can be evaluated as follows:

(14) $$T_{min}=\frac{\left(E_{max}-E_{min}\right)}{P_{max}}$$

(15) $$T_{max}=\frac{\left(E_{max}-E_{min}\right)}{P_{min}}$$

Thus, if the nodes are fully charged, it will take *T_min_* time before the first charging request is sent and *T_max_* before the last charging request is sent.

To demonstrate the link between the solutions to the two challenges, the proposed strategy initially sets the WMCD at the site of the base station. Vacation refers to the state in which the mobile recharger is stationed at the sink node. During the vacation state, the mobile recharger:

(i) Recharges its battery;
(ii) Replaces its battery;
(iii) Becomes aware of the received recharging requests.

Denote g as the number of sets to be classified, which is set up as follows:

(16) $$g=⎡log_{2}⎣\frac{T_{max}}{T}⎦⎤$$

During this phase, we establish the charging period *T_i_* for every node *i* and categorize the set *Z_k_*. To begin, we set each node *i* (*i* ∈ N) ‘s charging period *T_i_* as:

(17) $$T_{i}=2^{a-1}*T\;\;\;\;\;\;\;\;\;\;\;\;\;\;\;\;\;\;\;\left(1\leq a\leq g\right)$$

Here, *a* is the approximate logarithm of the *T_i_* and *T* ratio, obtained as follows:

(18) $$a=⎣log_{2}\left(\frac{E_{max}-E}{p_{i}*T}-1\right)⎦+1$$

We define set *Z_k_* (1 ≤ *k* ≤ *g*) and let *i* ∈ *Za*; the WMCD will visit node *i* in the ($n\times 2^{a-1}$)th trip cycle. During the Lth cycle and the design of the WMCD’s travel path, we can obtain *F_j_*, which is the set of sensor nodes that should be charged in the *L*th cycle. We can express *L* (1 ≤ *L* ≤ 2^g−1^) as *j* = *n*. 2^c^, where *n* is an odd number and can integer and, here, *c* ≥ 0.

(19) $$F_{j}=\left\{\begin{array}{ll} Z_{1} & \left(c=0\right)\\ Z_{1}\cup Z_{2}\cup Z_{3}\ldots ..Z_{c+1} & \left(c\geq 0\right) \end{array}\right.$$

The steps explained above are given in the Algorithm 1 below.
**Algorithm 1.** ERMS procedures.**ERMS algorithm**1.Determine the value of *T* and the number of the visits set2.Initialize *P_max_* and *P_min_*3.Initialize *e_min_* and *e_thresh_*4.Set *g*5.Set the recharging period of node *i*, *T_i_* and classify *Z_k_*6.Define *Z_1_*, *Z_2_*, …, *Z_g_*7.For *I* = 1, 2, 3,…, *n* do8.  a = $⎣log_{2}\left(\frac{E_{max}-E}{p_{i}*T}-1\right)⎦+1$
9.   *I*
∈
*Za*  $T_{i}=2^{a-1}*T$10.End for11.Set the visiting nodes an and traveling path of *T*12.For *j* = 1, 2, 3,…, $2^{a-1}$13.   If *j* is odd, then14.   *F_j_* = $Z_{1}$15.  else16.   *F_j_* = $Z_{1}\cup Z_{2}\cup Z_{3}\ldots ..Z_{c+1}$17.  End if18.  For ∀$n_{i}$ ∈ *F_j_*
**do**19.   Charge nodes $n_{i}$ to *E_max_*20.  End for21.End for

### 4.2. Particle Swarm Optimization (PSO)

The PSO is made up of particles, which are members of the population. Each particle represents a bird in a flock, a swarm of fish or a potential result of an optimization problem. PSO starts by setting with (nPop) the number of particles and every particle in an *n*-dimension solution space. The autocorrelation of particle positions, the average velocity of each particle per iteration and the fitness of the search are three factors that describe the movement pattern in a particle swarm. These variables represent swarm movement patterns and how they affect inertia weight and velocity coefficients (ω and *c*).

The PSO algorithm’s general steps are described below, with the input number of particles m, n number of dimensions of the solution space (*dim*), inertia weight ω, learning coefficients $c_{1}$ and $c_{2}$ with cognitive and social components, respectively.

Step 1: In the n-dimensional space, create m particles and assign each one an initial position $X_{i}$ and an initial velocity $V_{i}$ In Equations (20) and (21), we define the position $X_{i}$ and the velocity $V_{i}$ vectors of the ith particle as follows:

(20) $$X_{i}=\left(X_{i,1},\;X_{i,2}\ldots ,\;X_{i,dim}\right)$$

(21) $$V_{i}=\left(V_{i,1},\;V_{i,2}\ldots ,\;V_{i,dim}\right)$$

Step 2: Based on each particle’s position, we calculate the fitness function f as shown in Equation (22). We define one cycle (Cyc) time as the interval between two charging requests, and one cycle time is a summation of the vacation time, the charging time and the travel time for all the nodes visited. Therefore, the number of cycles can be defined as the number of charging requests received during the lifetime (total time) of the network.

(22) f=Cyc102⎣log10Cyc⎦−1−82+emin−1−82+ethresh−1−82+1tvac−1−82+(Dtotal102⎣log10Dtotal⎦−1−8)2

$$s.t\;\;\;\;\;\;\;\;\;E_{min}<e_{min}<e_{thresh},\;\;\;\;\;\;E_{min}=0.05*E_{max}\;e_{min}<e_{thresh}<E_{max},\;\;\;\;\;\;\;E_{max}=10.8\;KJ$$

where Cyc is the time as the interval between two charging requests and $D_{total}$ is the total distance traveled by the WMCD throughout all cycles.

Step 3: Update each particle’s velocity and position as follows:

Every iteration, the position $X_{i}$ and velocity $V_{i}$ of each particle in the swarm are updated through Equations (23) and (24) as follows:

(23) $$V_{i}\left(iter+1\right)=\omega \cdot r\cdot V_{i}\left(iter\right)+c_{1}\cdot r_{1}\left(PBest_{i}\left(iter\right)\right)-X_{i}\left(iter\right)\;+c_{2}\cdot r_{2}(Gbest_{i}\left(iter\right)-X_{i}\left(iter)\right)$$

(24) $$X_{i}\left(iter+1\right)=X_{i}\left(iter\right)+V_{i}\left(iter+1\right)$$

where *r_j_* is a number between [0, 1] and *j* [0, 1, 2], generated randomly. Every iteration, the particles in the swarm move closer to the solution of the problem and update their positions towards *PBest* and *GBest*. Equations (23) and (24) generate additional position and velocity vectors for the next iteration (iter + 1). The inertia weight ω is a learning coefficient associated with the previous velocity, while learning coefficients $c_{1}$ and $c_{2}$ are associated with the cognitive and social components, respectively.

Step 4: Then, we check if the ending condition has been met; the algorithm is terminated if this is the case; otherwise, the algorithm goes back to Step 2. The PSO ending condition could be that it has completed the maximum number of iterations or that the global optimal position’s fitness function value does not increase by a certain amount. In Equations (25) and (26), the *PBest* position attained by the *i*th particle and the *GBest* position of the swarm is defined as:

(25) $$PBest_{i}=\left(pbest_{i,1},\;pbest_{i,2},\;\ldots ,\;pbest_{i,dim}\right)$$

(26) $$GBest_{i}=min\;\left(PBest_{1},\;PBest_{2},\ldots ,\;PBest_{nPop}\right)$$

**Figure 3:**
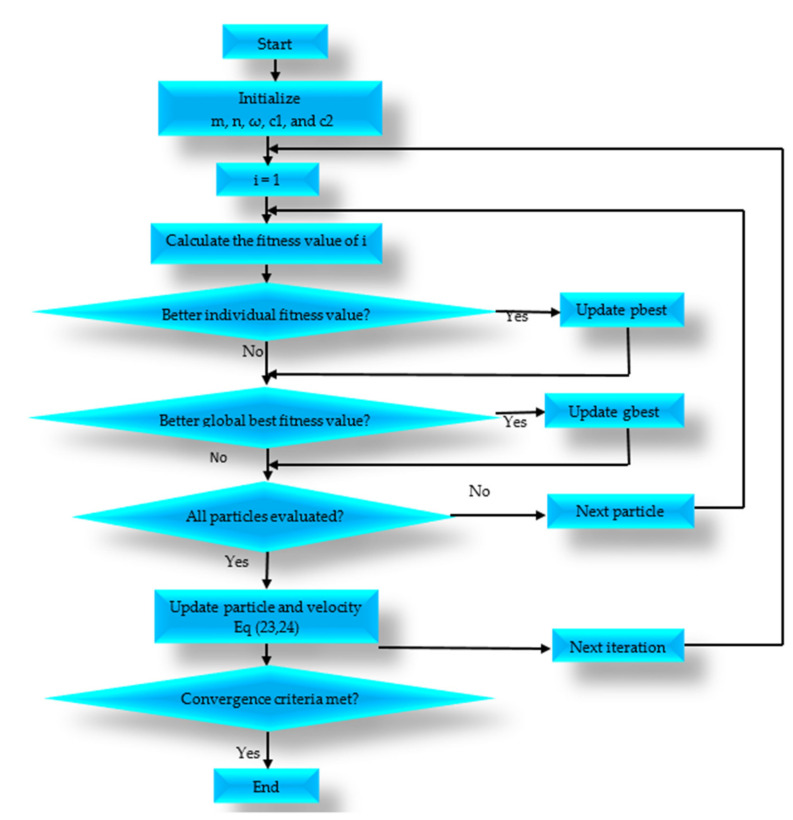
PSO flow chart.

The particle swarm optimization flow chart used in this work is shown in Figure 3.

## 5. Results

We present some numerical results in this section that show how ERMS-PSO performs in a real-world network. To evaluate the performance of the proposed ERMS-PSO, we used a network topology and parameter settings similar to those in [13,14,15,16], except for the wireless network size, which we compared to different wireless network sizes. The simulations were run using Matlab software. The parameters used in this study are shown in Table 1.

**Figure 4:**
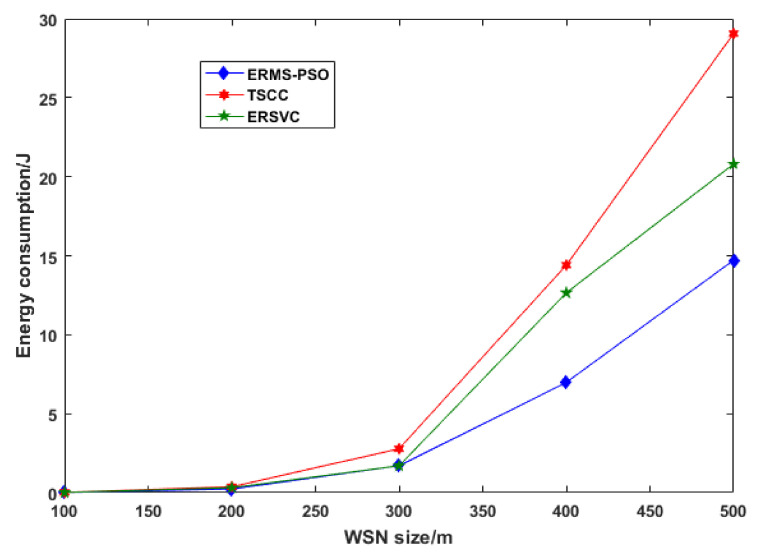
Energy consumption.

**Figure 5:**
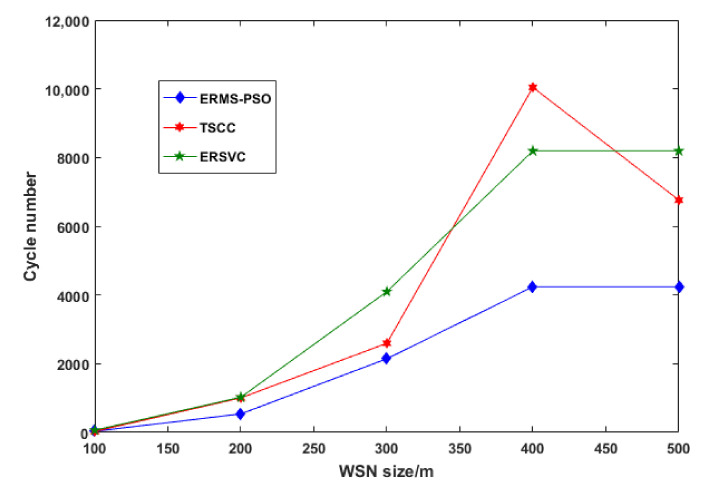
ERMS-PSO running cycles.

We run the results for the 50-node network. We compared our strategy with the traditional scheme with a constant cycle (TSCC) and an energy-efficient renewable scheme with a variable cycle (ERSVC). Table 2 shows the location and data rate of each node in a (100 × 100) m^2^ network. Figure 4 depicts the energy consumption results after the first step of ERMS-PSO optimization. These results compare the proposed energy renewable management system based on particle swarm optimization. As we can observe, for the five different network sizes used, ERMS-PSO consumed less energy than other schemes. Figure 5 shows the cycle number; each scheme runs from 100 to 500 m in network size.

**Figure 6:**
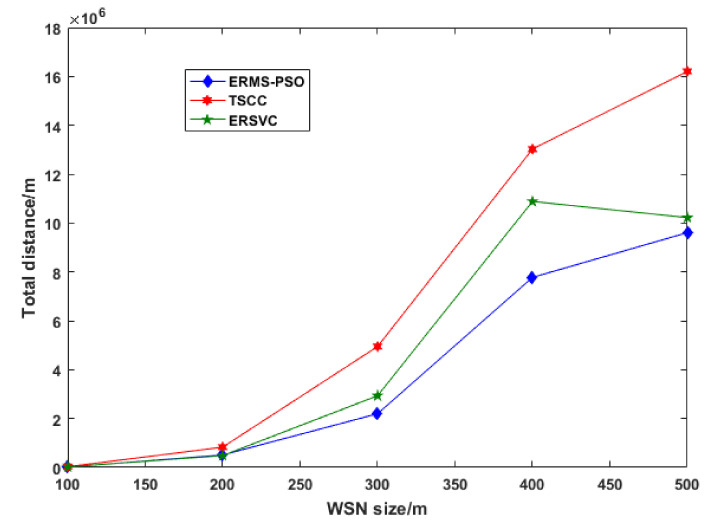
WMCD travel distance.

**Figure 7:**
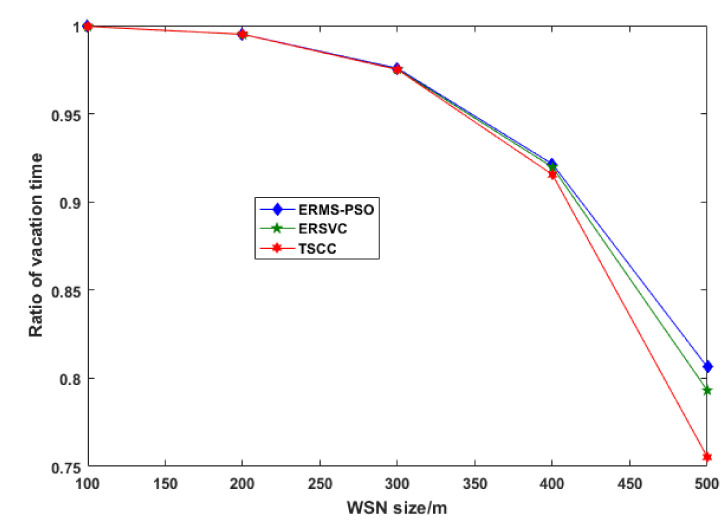
Ratio of vacation time.

As we can see from the above two figures, the fewer cycles that are run, the less energy consumed. Figure 6 shows the distance traveled by wireless mobile charging devices while charging nodes in a cycle. Our scheme outperforms others by using the shortest distance traveled by the WMCD, and this means that the distance is one of the most important issues to address because it contributes to the energy consumption. The more the WMCD travels, the more energy consumption and the less vacation time. Figure 7 shows the ratio of WMCD vacation time, and, as we can see, in our strategy, the vacation time decrease as the network area grows: for 500 m × 500 m, we achieved 81.6% compared to other strategies, where ERSCV had a value of 79.3% and TCSS had a value of 75.6%.

## 6. Conclusions

In this paper, we studied the operation of a sensor network based on WET, in a scenario where a WMCD was employed to charge the sensor nodes wirelessly inside the network. We analyzed the energy consumption of the entire system and pointed out that the traveling distance of WMCD is the main factor influencing total energy consumption. Based on energy consumption analysis and periodic strategy, we proposed a new new strategy called ERMS-PSO to decrease total energy consumption, travel distance and the charging number of cycles while maintaining the network operational with low complexity. In this new strategy, we introduced two sets of energies called emin (our minimum energy) and ethresh (our threshold energy), where, when the first node reaches our emin, it will inform the base station; then, the base station will calculate all nodes that fall under ethresh and send a WMCD to change them in one cycle. Our emin must be greater than the Emin (general minimum energy in the node) and our ethresh must be greater than emin but less than the general Emax. In contrast to previous schemes in which the WMCD visited and charged all nodes in each cycle, the WMCD in ERMS-PSO only needs to visit a subset of nodes by accounting for the difference in energy consumption rates at each node. As a result, the WMCD’s travel distance and total energy consumption can be significantly reduced. In ERMS-PSO, we first devised a system for combining each node’s charging period, the visiting set and the traveling trail during every cycle by first formulating a practical optimization problem with a flow rate to determine the energy consumption rate. Following this, we showed how ERMS-PSO can keep the network running. Based on simulations, ERMS-PSO can significantly reduce the total energy consumption while maintaining vacation time ratio performance. For future work, the ERMS-PSO strategy can be tested using a variety of new heuristic algorithms to determine which one performs the best and thus extends the the network life time.

## Figures and Tables

**Table 1:** Parameters used.

Simulation Parameters	Description of the Abbreviation
Nodes	50
Area length and width	100, 200, 300, 400, 500 m
RS, BS	center
*U*	5 W
*V*	5 m/s
*λ*	0.85
Electricity quantity	2.5 Ah
*E_max_*	10.8 KJ
*E_min_*	0.05 × *E_max_*
Data rate *R_i_*	[1, 10] kb/s
*β*_1_	50 nJ/b
*β*_2_	0.0013 pJ/b/m^4^
*α*	4
*ρ*	50 nJ/b
Inertia weight ω	2.1
Cognitive coefficient *c*_1_	2.24
Social coefficient *c*_2_	1.8
Number of particles *m*	20
PSO iterations	50

**Table 2:** Location and data rate for each node in a 50-node network.

Node Index	Location (m)	Data Rate (kb/s)	Node Index	Location (m)	Data Rate (kb/s)	Node Index	Location (m)	Data Rate (kb/s)
1	(42, 20)	5	18	(67, 26)	3	35	(40, 85)	9
2	(27, 61)	3	19	(92, 68)	4	36	(76, 43)	3
3	(76, 2)	2	20	(58, 58)	1	37	(58, 40)	9
4	(43, 72)	3	21	(29, 81)	7	38	(35, 35)	4
5	(22, 93)	8	22	(32, 47)	6	39	(29, 69)	7
6	(53, 74)	4	23	(22, 15)	8	40	(75, 96)	6
7	(49, 91)	7	24	(91, 43)	10	41	(65, 50)	10
8	(20, 40)	8	25	(92, 82)	6	42	(18, 26)	6
9	(94, 28)	2	26	(76, 65)	6	43	(28, 9)	8
10	(17, 78)	7	27	(6, 96)	5	44	(70, 58)	3
11	(92, 96)	1	28	(7, 52)	10	45	(61, 7)	2
12	(93, 14)	5	29	(46, 4)	9	46	(3, 81)	7
13	(79, 30)	8	30	(66, 79)	9	47	(4, 34)	5
14	(8, 21)	3	31	(86, 7)	6	48	(47, 62)	2
15	(87, 57)	10	32	(57, 84)	7	49	(64, 19)	2
16	(55, 28)	8	33	(17, 68)	3	50	(9, 7)	3
17	(9, 72)	5	34	(31, 93)	8			

## Abbreviations

**Abbreviations**	**Description**
*λ*	The efficiency of non-radiative energy transfer
*T*	WMCD periodic trip cycle
*H_n_*	Set of nodes that must be visited during the Cth cycle
*E_min_*	General minimum energy in the node
*E_max_*	Maximum energy in the node
*e_min_*	Proposed minimum energy
*e_thresh_*	Proposed threshold energy
*N*	Number of sensor nodes
*RS*	Rest station
*BS*	Base station
*R_i_*	Node *i* data rate
*p_i_*	Energy consumption rate at sensor node *i*
*WMCD*	Wireless mobile charging device
*P_k_*	Traveling path of WMCD
*D_n_*	Distance of Pk
*t_n_*	Time spent for traveling Pk
*t_vac_*	Vacation time of WMCD at rest station
*µ_vac_*	WMCD vacation time ratio
*WSN*	Wireless sensor network
*WET*	Wireless energy transfer
*ERSVC*	Energy-efficient renewable scheme with variable cycle
*TSP*	Traveling salesman problem
*ECPM*	Energy consumption per mile
*t_i_*	Charging duration of node *i*
*Pt_otal_*	System total energy consumption
*D_total_*	Total distance traveled over all cycles
*T_total_*	Total time spent over all cycles
*W_ij_*, *W_iB_*	Flow rate coefficient from node *i* to node *j* (or base station)
*V_ij_*, *V_iB_*	Energy consumption for transmitting a unit of data from node *i* to node *j* or base station
*ρ*	Constant coefficient
α	Path loss index
*d_ij_*	Distance between sensor *i* and sensor *j* (or base station B)
*β*_1_ and *β*_2_	Constant coefficients in transmission energy modeling
(*X_B_*, *Y_B_*)	Coordinates of the base station
*V*	Traveling speed of MCV
*U*	Energy transfer rate of MCV
*g*	The number of sets needing to be classified
*Z_k_*	The defined set that needs to be classified
*F_j_*	The set of nodes that should be visited during the *j*th cycle
